# First molecular data on the human roundworm *Ascaris lumbricoides* species complex from the Bronze and Iron Age in Hallstatt, Austria

**DOI:** 10.1038/s41598-023-38989-8

**Published:** 2023-07-25

**Authors:** Elisabeth Barsch, Kerstin Kowarik, Katharina Rodler, Christoph Hörweg, Hans Reschreiter, Helmut Sattmann, Julia Walochnik

**Affiliations:** 1 Institute of Specific Prophylaxis and Tropical Medicine, Medical University of Vienna, Vienna, Austria; 2 Prehistoric Department, Natural History Museum Vienna, Vienna, Austria; 3 3rd Zoological Department, Natural History Museum Vienna, Vienna, Austria; 4 Austrian Archaeological Institute, Austrian Academy of Sciences, Vienna, Austria

**Keywords:** Parasitology, Infectious diseases, Palaeontology

## Abstract

Palaeoparasitological studies can provide valuable information on the emergence, distribution, and elimination of parasites during a particular time in the past. In the prehistoric salt mines of Hallstatt, located in the Austrian Alps, human faeces have been conserved in salt. The aim of this study was to recover ancient DNA of intestinal parasites from these coprolites. Altogether, 35 coprolites from the Hallstatt salt mines, dating back to the Bronze Age mining phase (1158–1063 BCE) and the Iron Age mining phase (750–662 BCE), respectively, were analysed by microscopy and molecular methods. In 91% of the coprolite samples, eggs of soil-transmitted helminths (STH), namely of *Trichuris* and/or *Ascaris* were detected by light microscopy. The *Ascaris* eggs were exceptionally well preserved. For further analysis, DNA was extracted from the palaeofaecal samples and species-specific primers targeting different genes were designed. While amplification of *Trichuris* DNA remained unsuccessful, sequence data of *A. lumbricoides* species complex were successfully obtained from 16 coprolites from three different genes, the mitochondrial cytochrome c oxidase subunit 1 gene (*cox1*), the mitochondrial cytochrome B gene (*cytB*) and the mitochondrial NADH dehydrogenase subunit 1 gene (*nadh1*). Importantly, these included two *Ascaris* sequences from a coprolite from the Bronze Age, which to the best of our knowledge are the first molecular data of this genus from this period.

## Introduction

Hallstatt is a small village idyllically situated at a lake of the same name in the Eastern Austrian Alps and it is one of the most important archaeological sites in the Old World being the type site for the early European Iron Age, the Hallstatt period. The region is a UNESCO World Heritage area and bears one of the oldest known salt mines in the world. Large-scale underground salt mining is evidenced here since the 14th cent. BCE (Bronze Age). Only four prehistoric salt mines are known worldwide—two of them are in Austria, in Hallstatt and Dürrnberg, the third one is in Chehrabad in North-Western Iran, and the fourth one is in Duzdağı in Azberbaijan^1^.

Palaeoparasitology is a comparably young discipline allowing the reconstruction of the geographical distribution of parasites and their hosts over time, thereby revealing data on migrations of ancient populations, domestication of animals, cultural habits, hygiene, medical practices and other aspects of life such as sanitation and waste management^2^. The pioneer of palaeoparasitology, Sir Marc Armand Ruffer, discovered calcified eggs of the blood fluke *Schistosoma haematobium* in the kidneys of two Egyptian mummies dated to 1250 to 1100 BC^3^. In 1944, Szidat reported the detection of eggs of *Trichuris trichiura*, the human whipworm, and *Ascaris lumbricoides.*, the human round worm, in a bog mummy from what was then East Prussia^4^ and in 1960, Callen and Cameron published a method for rehydrating desiccated ancient material using 0.5% trisodium phosphate aqueous solution^5^. With this method it became possible to also study parasites from ancient faecal materials (coprolites). In Austria, the first palaeoparasitological studies were performed in the early 1970s on coprolites from the ancient salt mines in Hallstatt and Dürrnberg, reporting the findings of eggs of *T. trichiura* and *A. lumbricoides*^6^.

The aim of this study was to recover ancient DNA of intestinal helminths from coprolites of different time periods from the ancient Hallstatt salt mines. The large organic inventories (e.g. tools, working materials) excavated in the mining areas are well investigated (technology, materials analytics)^7^, but molecular data are still scarce and open up great potential. For the current study, coprolites were obtained under sterile conditions from different sites within the mining areas corresponding to different time periods and compared to archived material. Novel primers were designed to amplify short fragments of three mitochondrial marker genes for identification. As ancient DNA is usually available only at extremely low concentrations^8^, mitochondrial markers were selected as PCR targets, mitochondrial DNA (mtDNA) being present in a much higher copy numbers than genomic DNA.

## Material and methods

### Sampling sites

**Figure 1:**
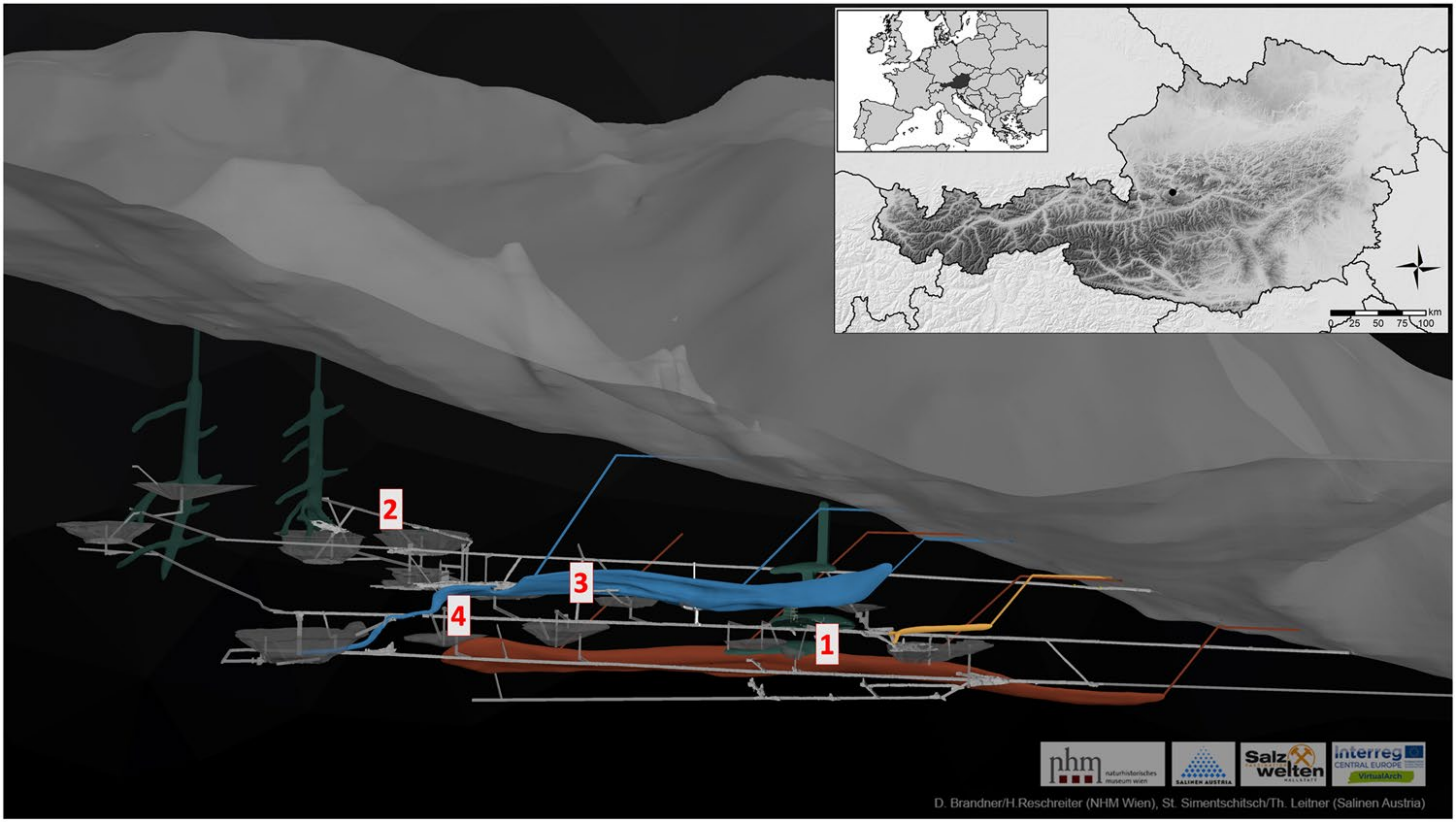
Cross-section showing the distribution of prehistoric mining sites in the Hallstatt salt mine. Green—Bronze Age, Blue—late Iron Age, Red—early Iron Age. 15—Kübeck (Aufdeckungsschlag) site, 5—Josef Ritschner Werk (Sinkwerksebentel) site, 2—Kernverwässerungswerk site, 8—Christian von Tuschwerk site (D. Brandner/NHM Vienna). Small window: Map showing the location of the site within Austria and Europe (generated by Julia Klammer).

**Figure 2:**
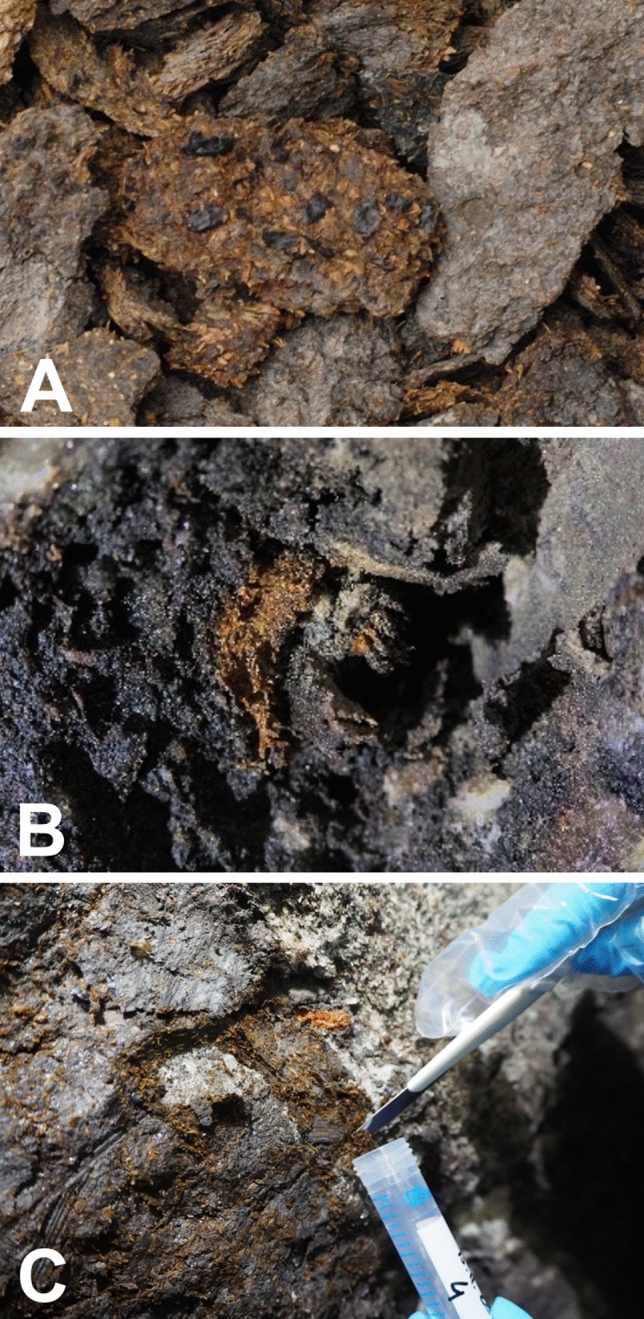
Coprolites embedded in soil. Coprolites can be distinguished from the surrounding material by their brownish colour and their amorphous texture. (**A**) archived coprolites. (**B**) fresh coprolites at the sampling site. (**C**) sampling after removal of surface layers.

The sampling sites included in this study were the late Bronze Age mining gallery located in the modern-day “Christian von Tuschwerk” site, as well as the early Iron Age sites “Kernverwässerungswerk”, “Kübeck” and “Josef Ritschner Werk” (Fig. 1). The sites in the Bronze Age mining areas, as well as early Iron Age mining areas are located more than 100 m below ground. The production debris of prehistoric mining activity, such as waste rock, left-over salt, burnt torches and broken tools, as well as the miners’ excrements remained in the mines and formed thick layers of waste. After the abandonment of the mine, mountain pressure re-closed all prehistoric mining chambers enclosing all materials left behind and compressing the debris layers to rock-like hardness. Due to the high salt concentration organic objects remained perfectly preserved over the millennia^7,9^. The Natural History Museum Vienna has been conducting excavations in the prehistoric mine workings since the 1960s. The mining phases have been dated by means of dendrochronology: The late Bronze Age “Christian von Tuschwerk” site dates to (1158–1063 BCE) and the early Iron Age sites mentioned above date to (750–662 BCE)^10^. The coprolites (Fig. 2) are embedded in the mining debris and can be found at various sites. Temperature and humidity are relatively constant all the year round and there is no natural light.

### Samples

In total, 35 coprolite samples from the ancient Hallstatt salt mine were analysed in this study. Four samples were from the late Bronze Age (12th to 11th cent. BCE) and 31 samples from the early Iron Age (8th to 5th cent. BCE). The samples were collected on five different dates several years apart (1989, 1993, 2003, 2015, 2018 and 2019). Twenty-six samples were from the collection of the Natural History Museum Vienna (Fig. 2A), four samples had been collected in 2018 for a previous study on coprolites from the Hallstatt salt mine^11^, and five additional samples were collected in 2019 for this study (Fig. 2B,C). These nine samples were collected under sterile conditions. First, the outermost layer was scraped off with a sterile scalpel and then the ancient faecal material was directly sampled into a sterile tube with a fresh sterile scalpel using double sterile disposable gloves and an FFP2 mask. During transport, the samples were refrigerated. In the laboratory, the samples were processed under sterile conditions in a laminar air flow cabinet (Heraeus HERAsafe^®^ HSP12, Kendro Laboratory Products GmbH, Hanau, Germany).

All samples were stored in individual, labelled plastic containers and opened and processed under sterile conditions. The colour of the coprolites was from greyish-light brown to dark brown (Fig. 2). The samples from the museum collection were dehydrated, the samples from 2018 and 2019 were moist and had a soft, clay-like texture.

### Microscopic detection

All samples were screened microscopically. For this, we tried and compared several protocols from the literature^12–15^ and established a modified protocol. In brief, aliquots of approximately 30 mg of each sample were transferred into a 1.5 ml Eppendorf tube and rehydrated in 0.5 ml 10% NaOH at room temperature by gentle shaking on a rocking platform overnight. On the following day, the samples were vortexed, and 40 μl of each suspension were placed onto a slide and investigated by light microscopy using a Nikon Eclipse E800 microscope (Nikon Instruments Inc., USA). Three slides per sample were analysed. Micrographs were taken with a microscope camera (Nikon DS-Fi2, Nikon Metrology, Germany) and estimations of egg numbers were made as described previously^6,15^. Rehydrated samples were stored at 4 °C.

### Single egg isolation

In order to prove that the eggs detected by microscopy still contained DNA, single eggs were isolated from samples with intact well-preserved eggs. For this, a drop of 100 μl dH_2_O was placed centrally into a sterile petri dish and 25 μl of the respective sample were added and mixed with the water. Single eggs were isolated with a micropipette (volume 5–10 μl) using an inverted microscope (Nikon TMS, Nikon Instruments Inc., USA). Single eggs were individually transferred to 1.5 ml tubes containing 50 μl of 70% ethanol for DNA extraction with the DNeasy^®^ PowerSoil^®^ Kit (QIAGEN, Hilden, Germany) as described below.

### DNA extraction

Altogether, 33 samples were selected for molecular analyses, from two of these we also had single eggs. As it is known that the yield of aDNA can vary significantly depending on the extraction protocol^16^, four different DNA extraction methods were evaluated and compared, including the QIAamp^®^ DNA Mini Kit (QIAGEN, Hilden, Germany), the QIAamp^®^ DNA Stool Kit (QIAGEN, Hilden, Germany), the GeneClean^®^ Kit for ancient DNA (MP Biomedicals, USA) and the DNeasy^®^ PowerSoil^®^ Kit (QIAGEN, Hilden, Germany). The latter, especially designed to remove inhibitors commonly found in soil and environmental samples, gave the highest DNA yields and was thus used for all samples. In brief, approximately 30 mg of rehydrated coprolite samples were processed with a Precellys^®^ 24 homogenizer (Bertin Instruments, France). Here, best results were obtained with 0.7 mm garnet beads (compared to 0.5 mm glass beads, 0.1 mm zirconium ceramic oxide beads, and a mix of these both). To remove as much NaOH as possible, samples were washed three times by transferring the samples with the beads to a fresh tube, adding 400 μl of sterile ddH_2_O, gentle vortexing and centrifugation at 20,000 × *g*/5 min. After washing, as much liquid as possible was removed, the pellet was resuspended in 200 µl sterile ddH_2_O and, together with the beads, transferred back to the PowerBead tubes. The samples were gently vortexed and then processed in the same way as the tubes with the single eggs by adding 60 μl of solution C1 (DNeasy^®^ PowerSoil^®^ Kit) and inverting the samples several times. Then, the samples were subjected to 3 cycles of homogenization at 5,800 rpm for 15 s with 60 s breaks in between and incubated overnight at 56 °C and 400 rpm. After vortexing and centrifugation at 10,000 × *g*/30 s, the supernatants were transferred to clean 2 ml collection tubes and washed according to the manufacturers’ instructions. The DNA was eluted in 20–30 μl Elution buffer, depending on the number of eggs in the sample, and stored at − 20 °C. Total DNA yields as measured by a NanoDrop™ spectrophotometer (ThermoFisher Scientific, Waltham, USA) ranged between roughly 50–500 ng/μl and were highest in the freshly obtained, non-dehydrated samples, as already shown in a previous study^11^.

### Primer design

Several primers from the literature, in-house primers and primers from a previous study^11^ were tested and compared. However, none of these primer pairs gave an amplicon of the correct size in the respective PCRs. Therefore, new primers for each species (Tables 1 and 2) were designed based on reference sequences from the NCBI database (https://www.ncbi.nlm.nih.gov/) and by using GeneDoc (National Resource for Biomedical Supercomputing, Pittsburgh, USA), ClustalX (Conway Institute, Dublin, Ireland) and OligoCalc (http://biotools.nubic.northwestern.edu/OligoCalc.html). The primers were designed to amplify short sequences with a length between 97 and 246 base pairs (bp), as ancient DNA usually is highly fragmented.

**Table 1:** Specific primers for amplification of *Ascaris* DNA.

Locus	Primer	Primer name	Primer sequence (5´–3´)	Melting temperature (°C)	Amplicon size (bp)
*cox1*	Forward	COX1FW_asc_EB	GGGTCTTTGGGTATGGTTTA	56.4	182
	Reverse	COX1RW_asc_EB	CCAAACAAGGTAGCCAACCA	58.4	
*cox1*	Forward	COX1FW2_asc_EB	CTGTTGGTATGGATCTTGA	53	172
	Reverse	COX1RW2_asc_EB	CGGTTAACCCACCAATAGTA	56.4	
*nadh1*	Forward	NADH1FW_asc_EB	GACTCCTTTGAATTCTTCGG	56.4	152
	Reverse	NADH1RW_asc_EB	GACTTTCTTACCATACCACT	54.3	
*cytB*	Forward	cytBFW_asc_EB	GTTAGGTTACCGTCTAGTAAG	57.5	97
	Reverse	cytBRW_asc_EB	CAAAAAGGCCAAAGCACCAT	56.4

**Table 2:** Specific primers for the identification of *Trichuris* DNA.

Locus	Primer	Primer name	Primer sequence (5´–3´)	Melting temperature (°C)	Amplicon size (bp)
SSU rRNA	Forward	18SFW_tri_EB	CTCGTAGTTGGATTGCGGAT	58.4	180
	Reverse	18SRW_tri_EB	CACCACGGTTCAAGCACTA	57.5	
SSU rRNA	Forward	18SFW2_tri_EB	GGAACGTTTCTCCATGAGAC	58.4	239
	Reverse	18SRW2_tri_EB	GAGCATCCAGGGCAATCTT	57.5	
SSU rRNA	Forward	18SFW3_tri_EB	CTAGTTGTGACCGTAAACGA	56.4	230
	Reverse	18SRW3_tri_EB	GATCTGTCAATCCTCGCAGT	58.4	
ITS2	Forward	ITS2FW_tri_EB	GTTGAAGAACGACGTGACAC	58.4	170
	Reverse	ITS2RW_tri_EB	CACCAACCTCAACCTGTAG	57.5	
ITS2	Forward	ITS2FW2_tri_EB	CTAGTAGCATTCGAACTGCT	56.4	201
	Reverse	ITS2RW2_tri_EB	GCTTAACGACCCTCAGACA	57.5	

For *Ascaris*, four specific primers pairs were designed, including two primer pairs targeting the mitochondrial cytochrome c oxidase subunit 1 gene (*cox1*) with overlapping amplicons. The first primer pair was slightly modified from the literature (ACF2 and ACR2)^12^ and flanks an approximately 182 bp long fragment, the second primer pair amplifies an approximately 172 bp long fragment. The third primer pair targets a ~ 193 bp region within the mitochondrial NADH dehydrogenase subunit 1 gene (*nadh1*) and the fourth primer pair targets the mitochondrial cytochrome B gene and results in a ~ 138 bp amplicon.

### Polymerase chain reactions (PCRs)

All amplifications were carried out in 50 μl reaction volumes and tested in independent setups with different DNA concentrations and using a Hot FIREPol DNA polymerase (5 U/μl, Solis BioDyne, Tartu, Estonia). All PCRs were run in a thermocycler (Eppendorf, Hamburg, Germany) starting with an initial denaturation step (hot start) at 95 °C for 15 min, followed by 35 cycles of 1 min at 95 °C, 45–50 s between 52 and 54 °C depending on primer and 50 s at 72 °C with a final elongation step at 72 °C for 7 min. The cycling conditions for all primer pairs are given in Tables 1 and 2. The PCR results were visualised by agarose gel electrophoresis using a transilluminator (Gel Doc XR +, Bio-Rad Laboratories, Inc., California, USA). Amplicons were stored at 4 °C for further use.

### DNA sequencing

All bands of appropriate sizes were excised from the gels with a sterile scalpel. DNA was purified from the bands using the GFX™ PCR DNA and Gel Band Purification Kit (Cytiva Europe GmbH, Wien, Austria) according to the manufacturer´s instructions. The DNA was eluted in 10–50 μl elution buffer depending on the intensity of the bands. Purified DNA was stored at − 20 °C. Sequences were obtained from both strands in at least two independent setups using the BigDyeTM Terminator v1.1 Cycle Sequencing Kit and a SeqStudio automated sequencer (Applied Biosystems, ThermoFisher Scientific, Waltham, USA). The obtained sequences were assembled to consensus sequences using GeneDoc and Chromas (Technelysium Pty Ltd., Australia) and compared to reference sequences using ClustalX. MEGA-X (Molecular Evolutionary Genetics Analysis, Pennsylvania State University, USA) was used for further data analysis.

## Results

### Microscopy

**Figure 3:**
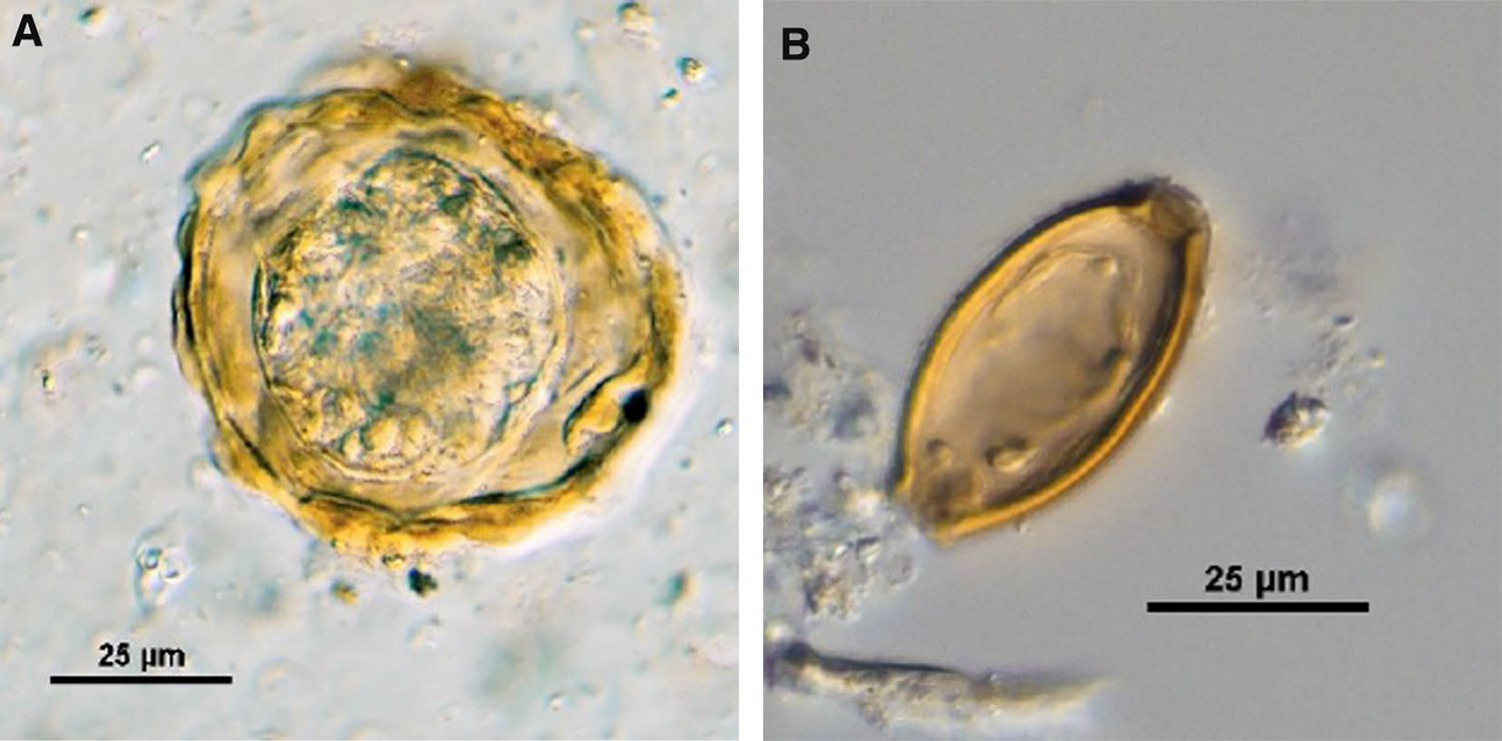
Micrographs of helminth eggs from coprolites. (**A**) *Ascaris* egg from a sample from the Bronze Age. (**B**) *Trichuris* egg from a sample from the Iron Age with damage on the egg shell. Interference contrast microscopy, magnification: 400 ×.

Altogether, 35 coprolite samples, four from the Bronze Age and 31 samples from the Iron Age, were analysed by microscopy after rehydration. Of these 35 samples, 32 (91%) were positive for eggs of intestinal helminths (Fig. 3). Eggs of *Trichuris* were detected most frequently, namely in 31 samples. Of these, 14 samples contained only eggs of *Trichuris* and 17 samples additionally contained *Ascaris* eggs. One sample contained only eggs of *Ascaris*. Helminth eggs were detected in three of the four coprolites from the Bronze Age, two samples (sample IDs: 3.4041 and 2019/1) containing only eggs of *Trichuris* and one sample (ID: 18.215) containing eggs of *Trichuris* and of *Ascaris*. As already described in previous microscopic studies on coprolites from these sites^6^, egg densities were comparably high with up to ~ 7000 eggs/g. Compared to *Trichuris* eggs, *Ascaris* eggs were exceptionally well preserved (see also Supplementary Fig. 1).

As based on morphology only, it is not possible to differentiate between eggs of *Trichuris trichiura* and *T. suis* and between *Ascaris lumbricoides* and *A. suum*, respectively, we refer to *Trichuris* eggs and *Ascaris* eggs here.

No eggs of other helminths were detected in any of the samples.

### PCR results

Specific PCRs were run with all 18 samples that were microscopically positive for *Ascaris* (of two of these we also had single eggs), and with 10 samples microscopically positive for *Trichuris* (see also Supplementary Table 1)*.* From 16 *Ascaris* samples, including one sample also as single egg, amplicons of the correct lengths were obtained; in some cases, fragments of more than one gene were successfully obtained. Of the *Trichuris* samples, three did give amplicons, however not of the appropriate size. For all other *Trichuris* samples, no bands were retrieved. All visible PCR products were excised from the respective gels for DNA sequencing.

### *Ascaris* specific PCRs

In total, 31 PCR runs resulted in amplicons of the correct lengths. Cytochrome c oxidase subunit 1 (*cox1*) amplicons were obtained from 15 samples, cytochrome B (*cytB*) amplicons from nine samples and NADH dehydrogenase subunit 1 (*nadh1*) amplicons from seven samples. All amplicons were subjected to Sanger sequencing. Reliable sequences were obtained from 29 amplicons, including 15 *cox1*, eight *cytB* and six *nadh1* sequences. Of the *cox1* sequences, 11 samples were of high quality and consensus sequences were generated from both strands. The sequences of the remaining four PCR amplicons were too short and/or had too many ambiguous bases and were therefore excluded from further analyses. The obtained *cox1* consensus sequences have a length of 142 bp. Sequence identities ranged from 129 to 141/142 bp (92.81% to 99.30%). Of the eight *cytB* sequences, five gave reliable consensus sequences with a length of 97 bp and identities ranging from 90 to 96/97 bp (93.75% to 98.97%). *Nadh1* sequences were obtained from six samples of which five were of good quality. The obtained consensus sequences had a length of 152 bp and sequence identities ranged from 136 to 148/152 bp (90.67–98.00%). For six samples, sequences of at least two different genes were obtained. With these fragments, the samples were reliably identified as *A. lumbricoides* species complex, those that had the highest number of unambiguous bases showed highest identity to *A. lumbricoides* species complex clade A and lowest identity to clade C.

## Discussion

This study provides the first two ancient DNA sequences of the intestinal parasite *A. lumbricoides* species complex from the Bronze Age. Moreover, several sequences of different genes of *A. lumbricoides* species complex of coprolites from the Iron Age were obtained. This study revealed a high detection frequency of soil-transmitted intestinal helminths in the coprolites of the Hallstatt miners of the Bronze and the Iron Age, however only eggs of *Trichuris* and *Ascaris* were found.

Overall, 91% (32/35) of the palaeofaecal samples investigated revealed eggs of intestinal helminths, with *Trichuris* sp. being most abundant, detected in 31 samples. Fourteen samples also contained eggs of *Ascaris* sp. and one sample only contained *Ascaris* eggs. Importantly, eggs of both genera were detected in coprolites from both, the Bronze and the Iron Age. Thus, based on the current archaeological dating of the sampling sites^10^, it can be stated with certainty that the Hallstatt miners were infested with whipworms and roundworms, at least in the period between 1,158 and 662 BC. The actual parasite burden of the prehistoric miners or even more so of the general population of Hallstatt is difficult to assess, however our results and also those of previous studies^6,11^ indicate that both helminths were rather common at the time. Light infestations with these worms usually cause no symptoms, but people with heavy infestations or children may suffer from abdominal pain, diarrhoea and rarely also more severe symptoms. As the worms are comparably large and excreted when they die, it can be assumed that the miners knew about them. Preserved leaves of butterbur (*Petasites officinalis* and *Petasites paradoxus*) have been found in the mines, which are still today used in folk medicine to treat abdominal pain^17^. Both worm species are soil-transmitted helminths (STH) without an intermediate host, adult female worms producing thousands of eggs daily that are shed with the human stool. The eggs have to mature in the soil into infective eggs, which are then taken up orally via contaminated hands or foods by the next human host. Depending on the external conditions, it takes around 3–6 weeks for the eggs to become infective. The occurrence of roundworms and whipworms is an indication of poor sanitation and hygiene^18^. In Hallstatt, particularly during the Iron Age, hygiene conditions in the mines were obviously low, as cooking, eating and defecation were accomplished at the same spots. This is corroborated by the fact that palaeofeces can be found distributed throughout the salt mines^6,7,10,11,19^. So far, only one larger pile of palaeofaeces was found in the mining areas, namely at the “Kernverwässerungswerk” site, which might indicate that this spot was used more than once. A constant temperature of 8 °C and comparably high humidity prevails inside the mines and prehistoric miners worked closely together in working groups^7,10,19^, thus conditions were favourable for survival and transmission of helminth eggs. *Ascaris* and *Trichuris* are among the most common helminths found in paleoparasitological samples, and together with the hookworms, they are still the most common helminths in humans today, although no longer endemic in most parts of Europe, including Austria. Worldwide, approximately 807-1,121 million people are infested with *Ascaris* and approximately 604–795 million with *Trichuris*^20^. Eggs of *T. trichiura* were also detected in intestinal samples of the famous frozen “Iceman” mummy found in the Ötztal Alps dated to the 1st half of the 4th mil. BC^21,22^. Interestingly, no eggs of other intestinal helminths were found in this study, particularly no eggs of parasites associated with the consumption of meat or fish. In a previous study on coprolites from this salt mine^11^, structures resembling eggs of *Taenia* sp. and *Dibothriocephalus latus* (= *Diphyllobothrium latum*), were found, but could not be reliably identified. In the other prehistoric Austrian salt mine, which is located in Dürrnberg (Salzburg) around 60 km from Hallstatt, eggs of *Fasciola hepatica*, *Dicrocoelium dendriticum* and *Taenia* sp. have been found together with eggs of *Ascaris* and *Trichuris* in coprolites^6,19,23^. The tapeworms *Taenia* spp. and *D. latus* are transmitted via infested pork/beef and fish, respectively, if consumed raw or undercooked. Archaeological and archaeozoological studies suggest, that already during the Bronze Age, not only salt production but also meat (mainly pork) processing and conservation was an important economic branch in Hallstatt^7,24^. Butchered and cut meat parts were delivered to the mining community and cured with salt-rich mining debris in large wooden basins. In addition, archaeozoological and molecular analyses evidence that meat from different animals (pork, goat, beef and mutton) was consumed^25,26^. Earlier studies on palaeofaeces and the investigation of ancient cooking utensils have revealed that the Iron Age miners typically consumed a stew that mainly consisted of millet, barley, beans and meat and was cooked for many hours^27^. However there is also molecular evidence for substantial complexity in the prehistoric miners diet including the consumption of fermented foods (blue cheese) and beverages (beer) in Iron Age^26^.

In this study, a total of 19 *A. lumbricoides* species complex-specific sequences from different genes were successfully amplified and sequenced. This is the first time that ancient *Ascaris* DNA has been recovered from coprolites dated to the Bronze Age in Austria. In a previous study, ancient *Ascaris* DNA was detected by PCR, but no reliable sequence data was obtained^11^. It is noteworthy, that in the current study, only DNA from *Ascaris* was successfully amplified, while *Trichuris* DNA was not, either from the Bronze Age, or from the Iron Age. All PCRs with *Trichuris*-specific primers targeting different genes were unsuccessful. Molecular analysis of ancient material is challenging, the rate of DNA degradation depends on various external and internal factors^28^. We hypothesize that the specifically thick-walled *Ascaris* eggs protected the DNA better than the plugged *Trichuris* eggs. Morphologically, the *Trichuris* eggs showed more damage than the *Ascaris* eggs, and most of the eggs had lost the polar plugs and thus the internal contents were fully exposed to the salt. In other archaeological sites (but without high salt content), aDNA of parasites has been detected even in the absence of microscopically visible parasitic structures^29,30^.

Two aDNA fragments of *A. lumbricoides* species complex were obtained from a coprolite dated to the Bronze Age between 1158 and 1063 BC^10^, including a 142 bp long *cox1* sequence and a 152 bp long fragment of the *nadh1* gene. While several studies report the recovery of ancient parasitic DNA from archaeological sites dated to the Iron Age and later^31–35^, there is only a limited number of reports on intestinal helminth remains from the Bronze Age worldwide, and even fewer with molecular data. In a recent study, eggs of *Dibothriocephalus* sp., *Trichuris* sp., *Dioctophyme renale* (a dog parasite, only very rarely affecting humans), *Echinostoma* sp. and *Capillaria* sp. were detected by digital light microscopy in coprolites from a Late Bronze Age Must Farm in wetlands in England, UK^36^. On the island of Kea, Greece, eggs of *Ascaris* sp. and *Trichuris* sp. were detected in soil sediment samples from the sacrum and iliac area of burials from the Neolithic to Byzantine period^37^. In Sardinia, Italy, eggs of *Ascaris* and *Trichuris* were found in Bronze Age pits and wells^38^. Earlier studies on Bronze Age samples from Europe include findings of *Ascaris*, *Trichuris*, *Ancylostoma duodenale* (one of the two human hookworms) and *Dicrocoelium dendriticum* (the lancet liver fluke)^39–41^. Outside Europe, parasite eggs from archaeological excavations dated to the Bronze Age have been reported particularly also from Iran^42–44^. In a burial of a Bronze Age cemetery (2600–2200 BC), eggs of *D. dendriticum* were found, which represent the oldest finding of this parasite in the Near East^42^. Findings of *D. dendriticum* eggs in human faeces indicate a close relationship between humans and herbivores, as the life cycle of this parasite includes sheep, snails and ants. In Tel Meggido, Israel, a poorly preserved *Trichuris* egg was detected during microscopic investigations of faecal material found near the remains of a large palace dated to the Late Bronze Age^45^. But all these data were obtained by microscopy only. In sediment samples from the Neolithic (5320–4980 BC) lakeside settlement of La Draga in Spain, DNA of *Ascaris* sp., *T. trichiura*, *E. vermicularis*, *T. saginata* and *D. dendriticum* has been detected by multiplex PCRs. Again, two taxa (*D. dendriticum* and *E. vermicularis*) were not detectable by microscopy, which however revealed also *Dibothriocephalus* sp., *Capillaria* sp., *Paramphistomum* sp. (parasite of ruminants) and *Macracanthorhynchus* sp. (animal parasite, affecting mostly pigs and wild boar)^30^. In 2018, eggs of *Toxascaris* sp. were detected in a mammalian carnivore coprolite from an archaeological site in Argentina dated to 14,981–14,552 BC, confirmed by ancient mitochondrial DNA analysis, which identified the parasite eggs as *Toxascaris leonina*, a parasite of dogs, cats and other carnivores that can also infest humans, representing the oldest molecular record of a parasite worldwide^46^.

Limitations of this study were that the sample set was comparably small, with only 35 coprolites included. Also, the samples had been collected at different time points and stored for different periods of time. Moreover, this study only attempted to amplify aDNA of those parasites, of which eggs were detected by microscopy. Currently available molecular data do not allow for a reliable differentiation of *A. lumbricoides* and *A. suum* and rather suggest that *Ascaris* infecting humans is a species complex^47,48^, we thus refered to *A. lumbricoides* species complex throughout.

In conclusion, this study presents the first successfully amplified and sequenced ancient *Ascaris* DNA sequences from the Bronze Age and also the first micrographs of intestinal helminth eggs from the Bronze Age in Austria. Additionally, several sequences of different genes of *A. lumbricoides* species complex were obtained from coprolites from the Iron Age. Microscopically, *Ascaris* and *Trichuris* were detected in 91% of the palaeofaecal samples, indicating a high frequency of these parasites in at least a part of the prehistoric population. Interestingly, however, only these two parasite species were found.

## Supplementary Information

Supplementary Figure 1.

Supplementary Figure 2.

Supplementary Figure 3.

Supplementary Figure 4.

Supplementary Information.

## Data Availability

All sequence data have been submitted to GenBank and are available under the following accession numbers: OQ721964-OQ721974 (*cox1*), OQ626561-OQ626564 (*cytB*), OQ626565-OQ626569 (*nadh1*). Details of the methodological procedures are available in the Master Thesis that derived from this work^49^. All other data generated are available through the authors upon request.
