# Supplementary figures and images for: First molecular data on the human roundworm Ascaris lumbricoides species complex from the Bronze and Iron Age in Hallstatt, Austria
Source: Sci Rep. 2023 Jul 25;13:12055. doi: 10.1038/s41598-023-38989-8 (PMC10368691; doi:10.1038/s41598-023-38989-8)

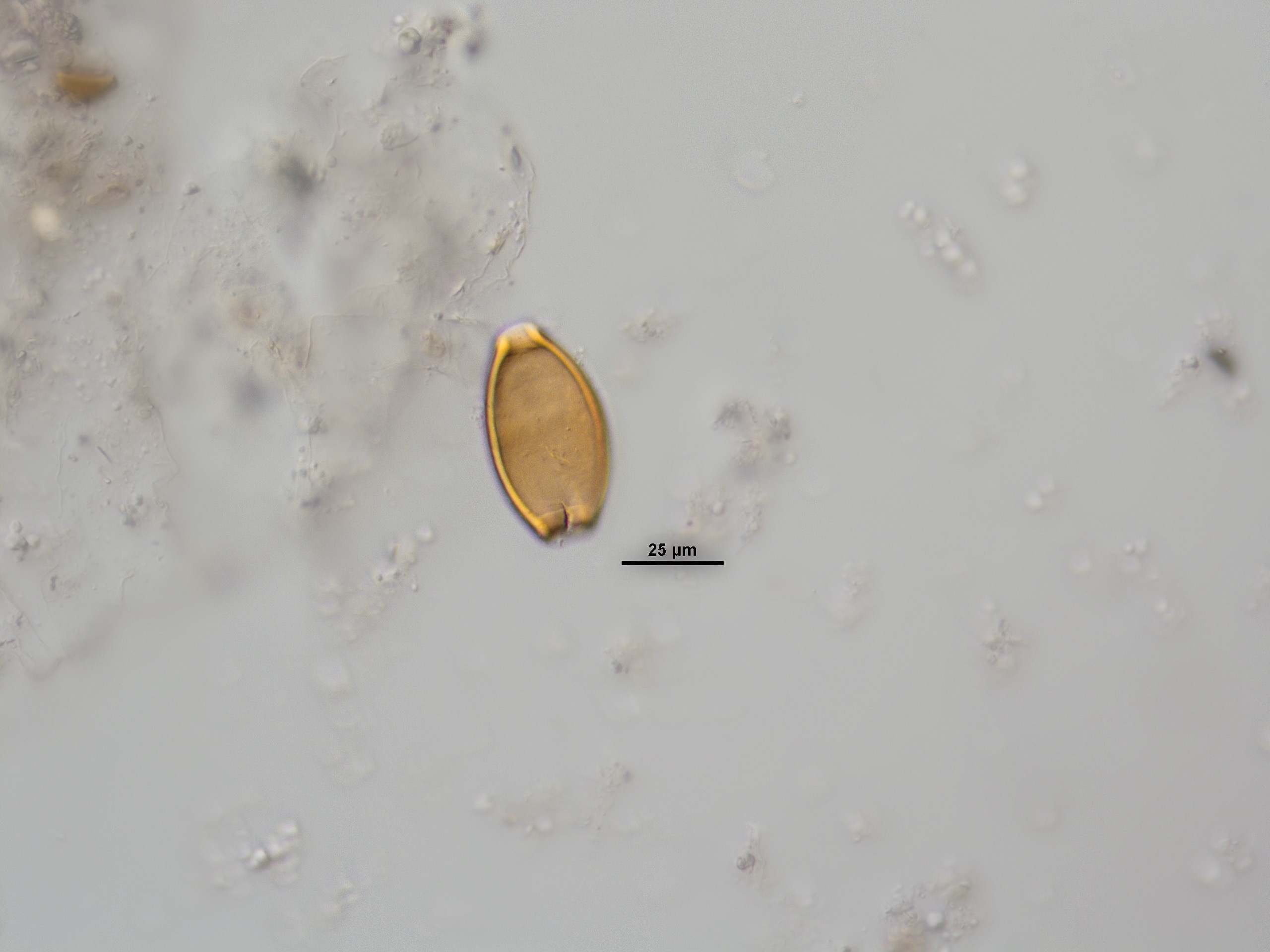

Supplement: Supplementary file 1 — Supplementary Figure 1. [file 41598_2023_38989_MOESM1_ESM.jpg]

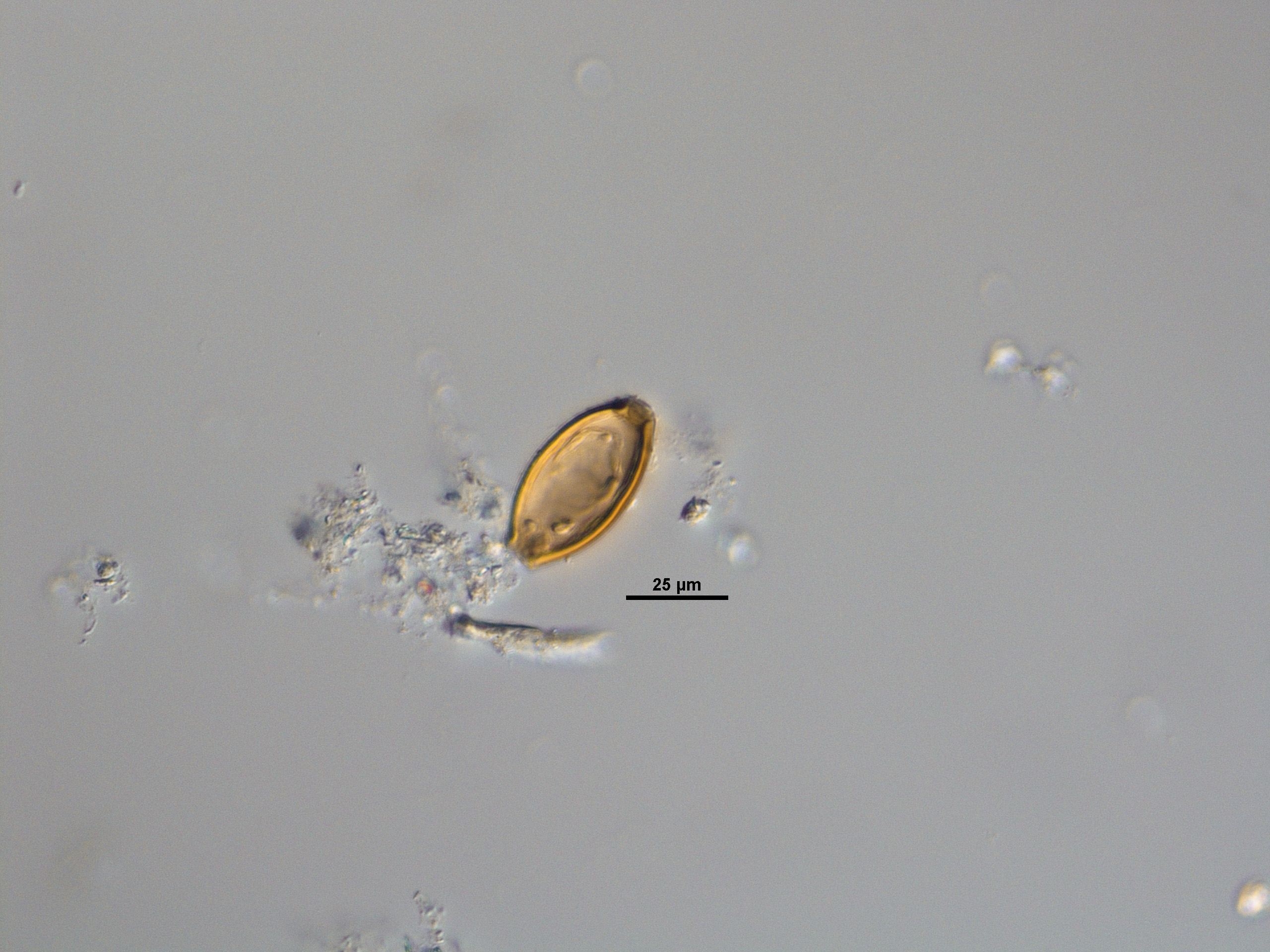

Supplement: Supplementary file 2 — Supplementary Figure 2. [file 41598_2023_38989_MOESM2_ESM.jpg]

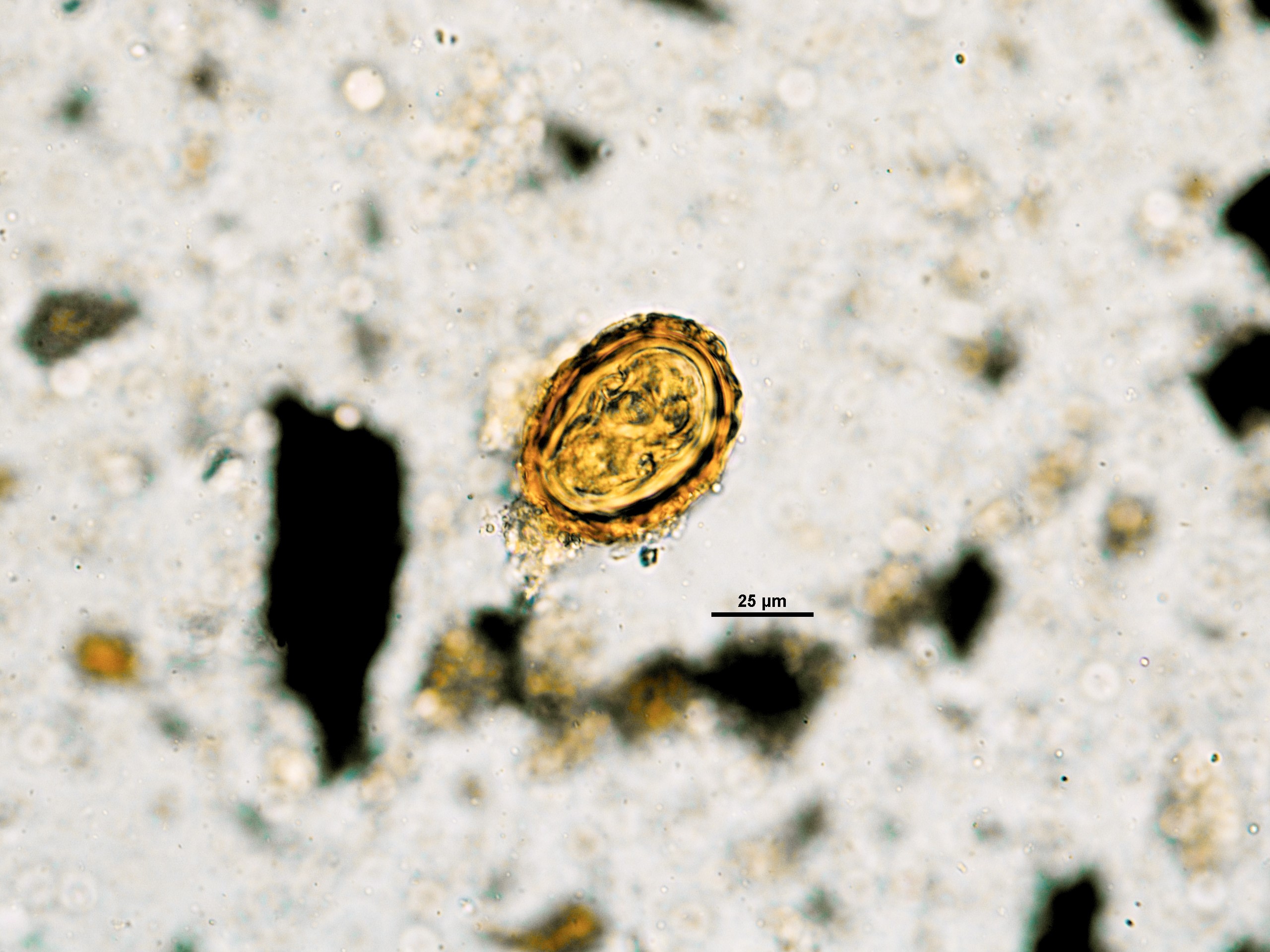

Supplement: Supplementary file 3 — Supplementary Figure 3. [file 41598_2023_38989_MOESM3_ESM.jpg]

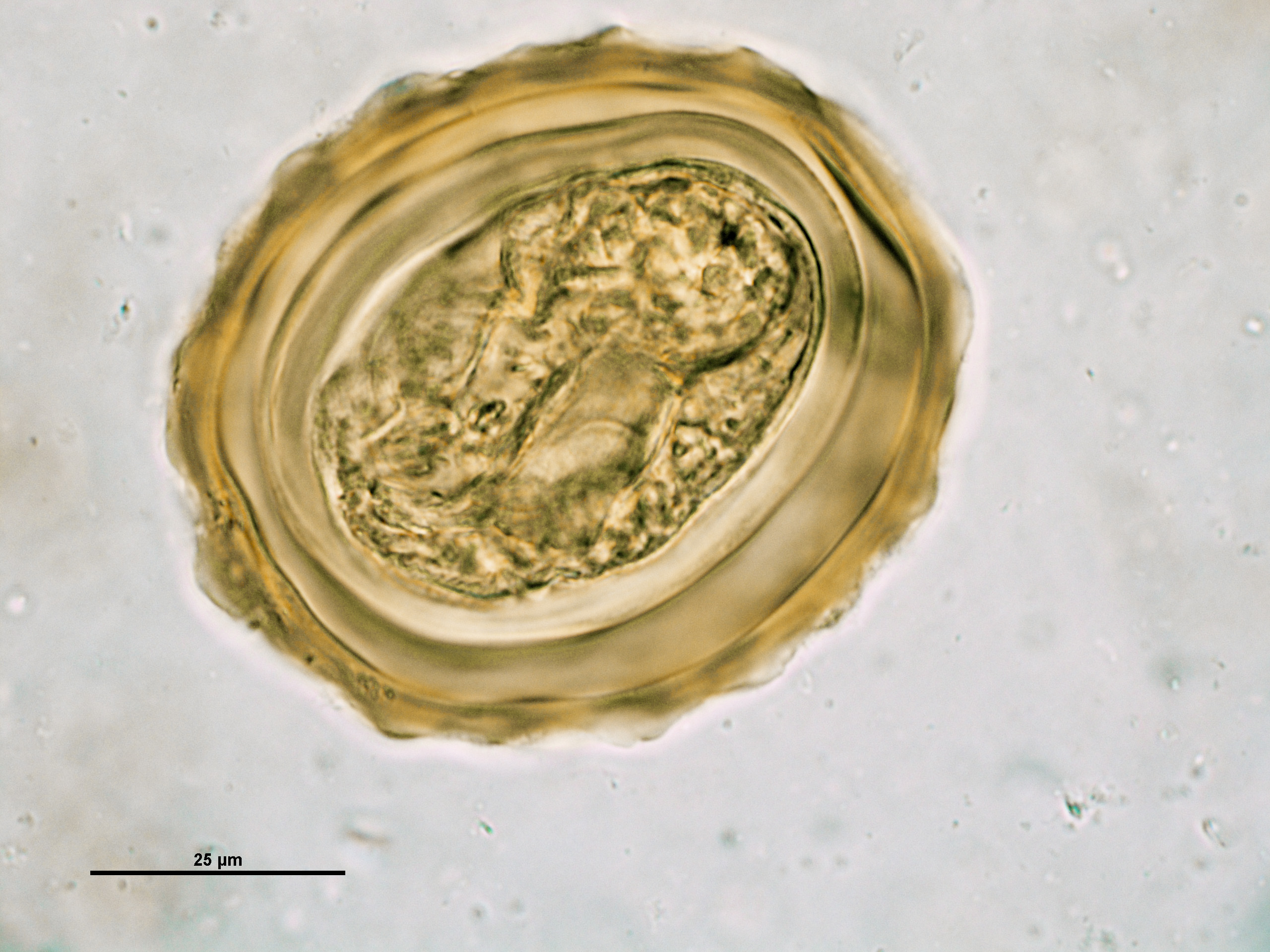

Supplement: Supplementary file 4 — Supplementary Figure 4. [file 41598_2023_38989_MOESM4_ESM.jpg]
