# Supplementary material for: First molecular data on the human roundworm Ascaris lumbricoides species complex from the Bronze and Iron Age in Hallstatt, Austria
Source: Sci Rep. 2023 Jul 25;13:12055. doi: 10.1038/s41598-023-38989-8 (PMC10368691; doi:10.1038/s41598-023-38989-8)
Supplement: Supplementary file 5 — Supplementary Information. [file 41598_2023_38989_MOESM5_ESM.docx]

| # | ID | Sampling  site | Sampling  date | Time period | *Ascaris lumbricoides* PCR | | | *Trichuris trichiura*  PCR |
| --- | --- | --- | --- | --- | --- | --- | --- | --- |
|  |  |  |  |  | *cox*1 | cytB | NADH1 |  |
| 1 | 89.826/5 | KW | 1989 | Iron Age | **●** | **x** | **x** |  |
| 2 | 89.826/6 | KW | 1989 | Iron Age | ● | ● | **x** |  |
| 3 | 90.213/1 | KW | 1993 | Iron Age |  |  |  | **x** |
| 4 | 90.213/2 | KW | 1993 | Iron Age |  | **x** | **●** | **x** |
| 5 | 90.213/3 | KW | 1993 | Iron Age | **●** | **●** | **x** | **x** |
| 6 | 90.213/4 | KW | 1993 | Iron Age |  |  |  |  |
| 7 | 90.213/8 | KW | 1993 | Iron Age | **●** | **x** | **x** |  |
| 8 | 90.213/9 | KW | 1993 | Iron Age | **x** | **x** | **x** |  |
| 9 | 90.213/10 | KW | 1993 | Iron Age |  |  |  |  |
| 10 | 90.245/1 | KW | 1993 | Iron Age |  |  |  |  |
| 11 | 3.041 | TW | 2003 | Bronze Age |  |  |  |  |
| 12 | OST 3.22 | TW | 2003 | Bronze Age |  |  |  |  |
| 13 | 121.28 3 | KW | 2015 | Iron Age |  |  |  | **x** |
| 14 | 121.30 2 | KW | 2015 | Iron Age | **●** | **●** |  | **x** |
| 15 | 121.81 | KW | 2015 | Iron Age | **x** |  |  |  |
| 16 | 121.86 6 | KW | 2015 | Iron Age | **●** | **●** |  | **x** |
| 17.1 | 90.213 A | KW | 1993 | Iron Age | **●** | **●** |  |  |
| 17.2 | 90.213 B | KW | 1993 | Iron Age |  |  |  |  |
| 17.3 | 90.213 C | KW | 1993 | Iron Age |  |  |  |  |
| 17.4 | 90.213 D | KW | 1993 | Iron Age |  |  |  |  |
| 17.5 | 90.213 E | KW | 1993 | Iron Age |  |  |  |  |
| 17.6 | 90.213 F | KW | 1993 | Iron Age |  |  |  |  |
| 17.7 | 90.213 G | KW | 1993 | Iron Age | **●** | **x** | **●** |  |
| 17.8 | 90.213 H | KW | 1993 | Iron Age | **●** | **●** | **●** |  |
| 17.9 | 90.213 I | KW | 1993 | Iron Age |  |  |  |  |
| 17.10 | 90.213 J | KW | 1993 | Iron Age |  |  |  |  |
| A | NN | KW | 2018 | Iron Age | **●** | **●** | **x** | **x** |
| B | 18.215 | TW | 2018 | Bronze Age | **●** | **x** | **●** |  |
| C | NN | KW | 2018 | Iron Age | **●** | **x** | **x** |  |
| D | NN | KB | 2018 | Iron Age | **●** | **x** | **●** |  |
| E | 2019/1 | TW | 2019 | Bronze Age |  |  |  | **x** |
| F | 2019/2 | JRW | 2019 | Iron Age | **●** | **●** | **●** |  |
| G | 2019/3 | JRW | 2019 | Iron Age | **●** | **●** | **●** |  |
| H | 2019/4 | KW | 2019 | Iron Age |  |  |  | **x** |
| I | 2019/5 | KW | 2019 | Iron Age |  |  |  | **x** |

**Supplementary Table 1**

Overview of the PCR results. Dots (●) indicate positive PCRs and crosses (x) indicate negative PCRs.

**Supplementary Figures**

Micrographs of *Ascaris* eggs and *Trichuris* eggs from coprolites.
